# A universal probe design for colorimetric detection of single-nucleotide variation with visible readout and high specificity

**DOI:** 10.1038/srep20257

**Published:** 2016-02-02

**Authors:** Xueping Chen, Dandan Zhou, Huawei Shen, Hui Chen, Wenli Feng, Guoming Xie

**Affiliations:** 1 Key Laboratory of Medical Diagnostics of Ministry of Education, Department of Laboratory Medicine, Chongqing Medical University, Chongqing 400016, P. R. China; 2 Clinical Laboratories, The First Affiliated Hospital of Chongqing Medical University, Chongqing 400016, P. R. China

## Abstract

Single-nucleotide variation (SNV) is a crucial biomarker for drug resistance-related detection in cancer and bacterial infection. However, the unintended binding of DNA probes limits the specificity of SNV detection, and the need for redesigned sequences compromise the universality of SNV assay. Herein, we demonstrated a universal and low-cost assay for the colorimetric discrimination of drug-resistance related point mutation. By the use of a universal DNA probe and a split G-quadruplex, the signal could be recognized by naked eye at room temperature. The DNA probe was used as a signal reporter which not only improved the universality, but also enabled high specificity of probe hybridization. This assay was successfully applied in the detection of cancer-related SNV in the epidermal growth factor receptor (EGFR) gene, kirsten rat sarcoma viral oncogene homologue (KRAS), and tuberculosis drug-resistance related point mutation in RNA polymerase beta subunit gene (rpoB) with high specificity and visible readout. This method was simple, rapid, high-throughput and effective, which was suitable for point-of-care applications.

Single nucleotide polymorphisms (SNPs) are DNA sequence polymorphisms resulting from a single nucleotide mutation at the genome leve^1^,^2^,^3^,^4^. SNPs are closely related to human diseases and drug efficiency^5^. In particular, single-nucleotide variants (SNV) at low variant allele frequency have been used as biomarkers for the early diagnosis of cancer^6^. Such as, point mutation of epidermal growth factor receptor 3 (EGFR 3) was highly expressed in bladder cancer, especially in low grade and superficial tumor^7^. It is important to select targeted drug toward epidermal growth factor receptor (EGFR) for effective treatment of colorectal cancer^8^. Kirsten rat sarcoma viral oncogene homologue (KRAS) is commonly expressed in kinds of cancers. 17–25% of all cancers have been proven to harbor mutations in KRAS, especially at codon 12 (35G>A and 35G>T)^9^. SNPs also have relationship with drug-resistance, for example, rifampin cannot combine with RNA polymerase B subunit when there are mutations in the core area, which lead to rifampin resistance. The detection of genetic phenotypes can be used for analysis of bacterial sensitivity to rifampin^10^,^11^. Thus, detections of SNPs are of great clinical significance. Conventional methods for SNP detection include SNP genotyping using MALDI mass spectrometry, allele-specific enzymatic amplification, probe-mediated clamping PCR, and real-time PCR^12^,^13^,^14^. But, these methods have some disadvantages, such as low detection efficiency, high cost and long analysis time^15^.

Therefore, simple and effective detection of SNV is of great importance in genomic studies and clinical diagnostics, and are urgently needed for the prescription of personalized medicine. To date, various technology platforms have been reported for detection of SNV^16^. A library of hybridization probes were designed and optimized properly for targeted mutated sequences in recent years^17^,^18^,^19^. Visual detection of SNV based on hairpin oligonucleotide-functionalized gold nanoparticle is reported by Liu and co-workers to reduce the execution time^20^,^21^. A self-complementary and triple-stem DNA probe has been developed by Soh *et al*.^22^. However, Specificity is the major drawback of these assays based on hybridization probes. Seelig and co-workers introduced the double-stranded toehold exchange mechanism to distinguish single base variation in target DNA^23^. Fluorescent DNA probes used in this method showed acceptable sensitivity and a wide linear range, but fluorescent modification is required and the format of nucleic acid detection is limited^24^,^25^. A highly sensitive and selective method for the detection of SNV based on peptide nucleic acid (PNA) is reported^26^,^27^,^28^. PNA can be applied to specifically recognize and bind complementary nucleic acid sequence, which greatly improve the diagnostic efficiency and sensitivity. However, the high cost of PNA probes and the careful manipulation in strict operating environment limit its application. It’s still a challenge to pursue novel, cost-efficient approaches to realize rapid and reliable identification of SNV.

In this work, we presented a universal strategy for the colorimetric detection of SNV with high specificity. This assay was single-step, enzyme-free, non-labelled and low cost. It was based on a well-designed DNA probe (X-probe) with a split G-quadruplex as the signal reporter. As depicted in Fig. 1, the X-probe was a conditionally colorimetric probe compromising four pre-hybridized strands: a split G-quadruplex A, a split G-quadruplex B, a specific protector strand P and a specific complement strand C. The two functionalized probe A and B have sequences decoupled from the SNV/WT sequences. Thus, the same A and B species could be used for kinds of X-probe designs and targeted different sequences. All of these probes were unmodified which largely decreased the cost. The reaction mechanism of X-probe and its intended target (SNV) was based on the toehold-mediated strand displacement. The toehold-mediated strand displacement is an enzyme-free process with excellent catalytic efficiency, which has been well-studied^29^,^30^,^31^. The hybridization between domain 10 and domain 11 led to the formation of G-quadruplex. Upon binding to hemin, the active-quadruplex/hemin complex exhibited superior peroxidase-like activity which could catalyze the conversion of a colorless 2, 2′-azinobis (3-ethylbenzothiozoline)-6-sulfonic acid (ABTS^2−^) to a green ABTS. Thus the colorimetric signal was generated. With one base mismatch, WT could barely initiate domain 4, and the G-quadruplex could not be split. Thus the absorbance was still high in the presence of WT. While in the presence of SNV, domain 4 known as the toehold was initiated, and then reaction between X-probe and SNV proceeded through a branch migration process. As a result, the AP subspecies were displaced, and the G-quadruplex was split, resulting in the decrease of colorimetric signal.

## Results and Discussion

**Figure 2:**
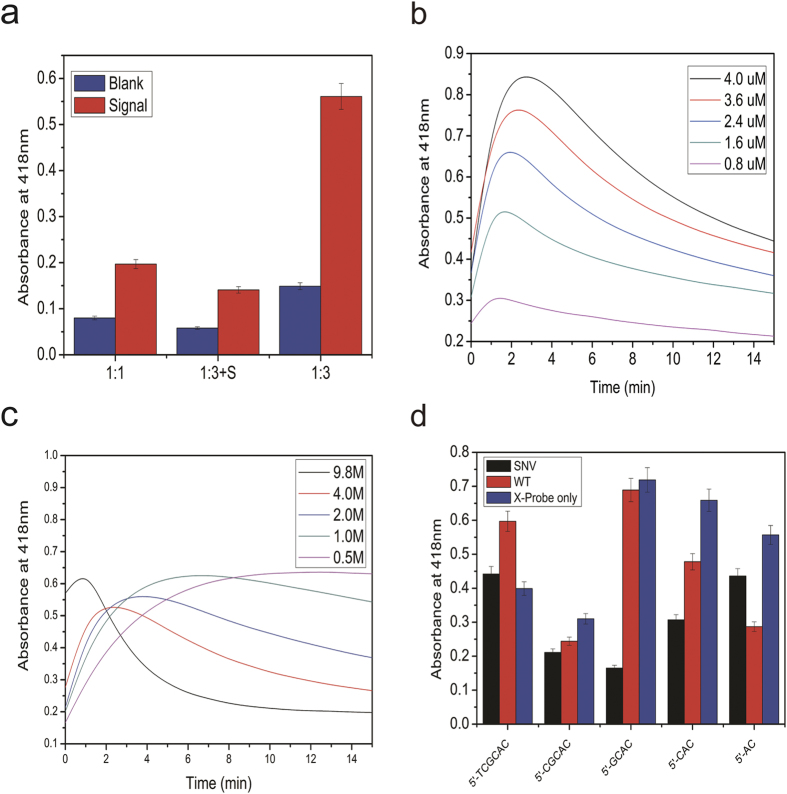
Optimizations of experimental parameters: the absorbance at 418 nm for (**a**) three kinds of G-quadruplex split modes (1:1, 1:3+s, 1:3). (**b**) Different concentration of hemin (0.8, 1.6, 2.4, 3.6, 4.0 μM). (**c**) Different concentration of H_2_O_2_ (0.5, 1.0, 2.0, 4.0, 9.8 M). (**d**) Five different sequences of signal strand A and B with different number of complement bases. The complement sequences were listed in Table S3. One parameter changed with the others under optimal conditions. The error bars were standard deviations of three repetitive measurements. UV–vis absorption spectra were obtained in the range from 400–500 nm.

In order to obtain ideal signal, the critical experimental conditions were optimized firstly. G-quadruplex split mode was the key point to obtain colorimetric signal. G-quadruplex with four GGG repeats can fold into a special four-stranded structure, which can dramatically enhance the catalytic ability of hemin^32^. Three kinds of G-quadruplex split mode were explored in this experiment. The four GGG repeats sequences were divided into two parts and designed in two seperate DNA probes. In the presence of target, the two seperate GGG repeats sequences could get close and led to the formation of G-quadruplex. In order to figure out the optimal way to split the G-quadruplex, three split modes were tested. The signal and blank of the three modes were shown in Fig. 2a. The signal of the 1:3 split mode which utilized an S strand for the formation of hemin-G-quadruplex DNAzyme was quite low^33^. For the two equally split mode (1:1)^34^, the signal was still not ideal. This might due to the formation of G:C in 1:1 and 1:3+S mode, which led to unintended secondary structure of signal probe. These secondary structures could hinder the formation of G-quadruplex. To avoid the unwanted structure, the sequence of G-rich segments was split into 1:3 mode without S probe. The signal of 3:1 split mode was obviously improved, and the signal to blank ratio was acceptable. So 1:3 mode was used in this method.

In order to optimize the concentration of hemin and H_2_O_2_, colorimetric signals were measured at different concentrations of hemin and H_2_O_2_. As shown in Fig. 2b, the absorbance changed along with different concentrations of hemin, when the concentration of hemin increased, the absorbance increased obviously. However, with the concentration increased, the background signal increased correspondingly. To balance the background and signal, 2.4 μM was used in our experiment. Figure 2c illustrated the effect of concentration of H_2_O_2_. The signal at 9.8 M increased and then decreased rapidly which was not stable for detection. In the range from 0.2 M to 2.0 M, the signal at 2.0 M reached highest at 2 min and remained stable for couple minutes. Thus 2.0 M was chosen as the optimized concentration of of H_2_O_2_ to obtain a stable signal with appropriate detection time.

In order to obtain higher specificity, we optimized the number of complement bases of signal strand A and B. we tested five different sequences (sequences are shown in supporting information). The detection signals of different sequence were provided in Fig. 2d. Among these sequences, the signal of SNV and WT could be discriminated obviously with sequence of 5′-GCAC. In one hand, more complementary bases between probe A and B was in favour of stable G-quadruplex, which resulted in higher signal. In other hand, it would hinder the cleavage between A and B, which had effect on discrimination between SNV and WT. To obtain higher specificity, sequence of 5′-GCAC in probe A was chosen.

**Figure 3:**
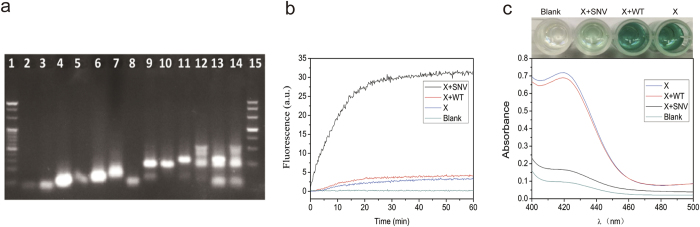
(**a**) 3% agarose gel electrophoresis images of Lane 1, 20bp DNA marker; Lane 2, SNV; Lane 3, WT; Lane 4, A; Lane 5, B; Lane 6, P; Lane 7, C; Lane 8, A+B; Lane 9, A +P; Lane 10, B+C; Lane 11, P+C. Lane12, A+P+B+C; Lane 13, A+P+B+C+SNV; Lane 14, A+P+B+C+WT. Lane 15, 500 bp DNA marker. The final concentration of DNA all probes were 2 μM. (**b**) The fluorescent characterization of blank, X-Probe, X+WT and X+SNV. Probe F and Q were modified with fluorophore and quencher respectively. The final concentration of each probe was 50 nM. (**c**) The absorbance at 418 nm of SNV, WT, and X-probe only. The final concentration of each probe was 200 nM. UV–vis absorption spectra were obtained in the range from 400 to 500 nm.

In order to prove the feasibility of the experiment, we did an agarose gel electrophoresis (Fig. 3a). Lane 1 to lane 7 presented the band of each single strand. Lane 8 to lane 11 were the bands of correspongding duplex strands. Lane 12 was the X-probe, which contained four strands (A:B:P:C complex) and some spared single strand or duplex. It showed that they could combine with each other to form the A:B:P:C complex with larger molecular. Lane 13 was added with SNV on the basis of lane 12. From the results of lane 13, we could know that SNV could replace P:C complex form A:B:P:C complex. However, WT has one different base compared with SNV, which could barely replace P and C as shown in lane 14. The above demonstrated the good feasibility and specificity of this method.

To investigate whether the method could be used for detection of SNV, the fluorescent X-probe was designed. It was consist of four chains that owed conditionally fluoresce on hybridization to its DNA target. The strand F and Q was modified with fluorescent and quencher, respectively. As shown in Fig. 3b, in the presence of target chain (SNV), Q:P complex could be replaced, and Q:F was split, leading to the strong fluorescence, shown in the black line of Fig. 3b. To prove the specific of the method, we used the WT chain which has only one mutant base. Due to the mutant base compared with SNV, the effect of strand displacement was greatly decreased and the signal of fluorescence was very low, as shown in the red line of Fig. 3b. Its fluorescence signal was as much as blank. In the results of colorimetic detection (Fig. 3c), the absorbance of SNV was as low as blank. The absorbance of WT was as high as X-probe only, which was obviously higher than the signal of SNV. And the results could be seen by naked eyes. These results demonstrated that this sensing strategy could be used for efficient discrimination of SNV with visible readout.

**Figure 4:**
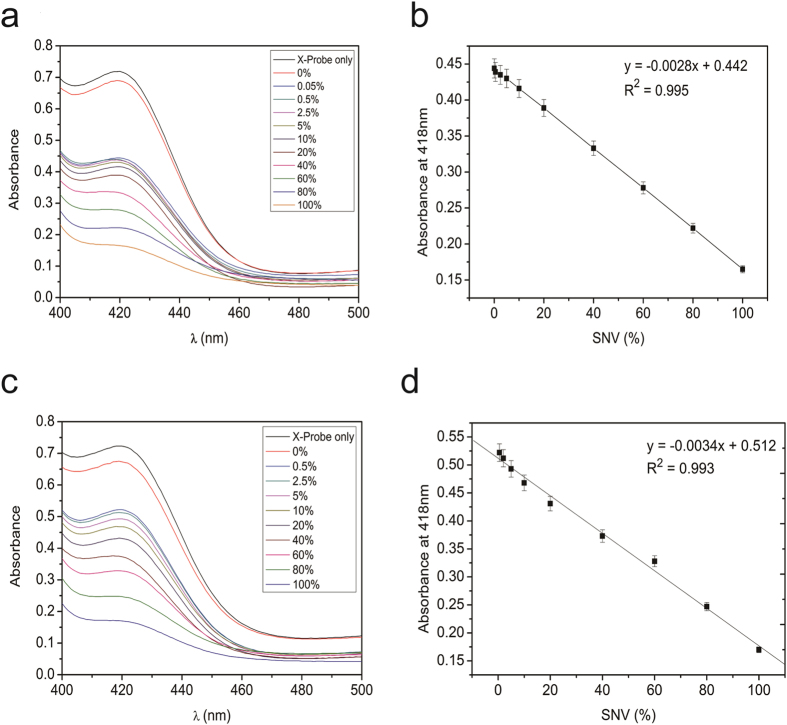
(**a**) The absorbance at 418 nm in the detection of SNV in KRAS-G12R at different abundances. (**b**) Relation of absorption peak with percentage of the SNV in KRAS-G12R. (**c**) The absorbance at 418 nm in the detection of SNV in rpoB-531 at different abundances. (**d**) Relation of absorption peak with percentage of the SNV in rpoB-531. 100% means the tested strands were all mutant type. 0% means the tested strands were all wild type. The error bars were standard deviations of three repetitive measurements. UV–vis absorption spectra were obtained in the range from 400 to 500 nm.

To demonstrate the application of this assay method targeting KRAS and rpoB, SNV were analyzed at different variant allele frequency (VAF) under the optimized conditions. As show in Fig. 4a,b, the mixtures containing KRAS-G12R mutant and its wild-type were used as the target. The absorbance decreased with the increment of KRAS-G12R mutant type percentage. A linear response toward the percentage of SNV from 0.05–100% was clearly obtained in the 96-well plate. The regression equation was y = −0.0028x + 0.442 with a correlation coefficient of 0.995. In addition, the same experiment was performed using rpoB-531 as the target. As it could be seen in Fig. 4c,d, the signal decreased with an increasing ratio of rpoB-531 mutant type. A linear response toward the percentage of SNV from 0.5–100% was clearly obtained. The regression equation was y = −0.0034x + 0.512 with a correlation coefficient of 0.993. This method also exhibited a good linear response toward rpoB-531 mutant type in the range from 0.5–100%. These results demonstrated that this sensing system showed good analytical performance toward the detection of SNV at different VAF, which might be suitable for the detection of different kinds of SNP.

**Figure 5:**
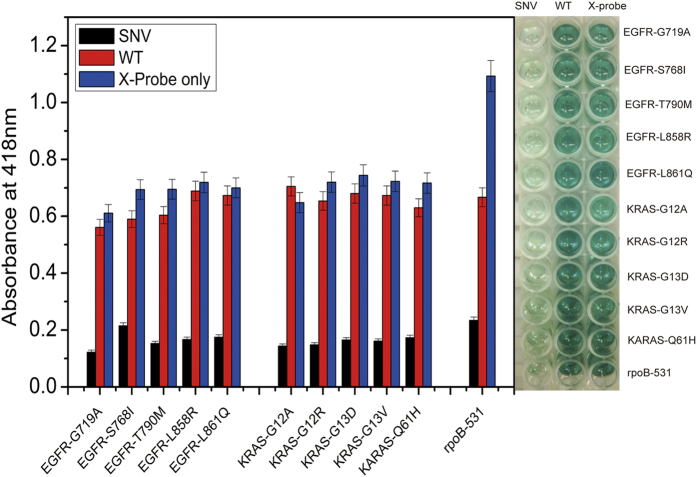
Summary of competitive composition experimental results of kinds of SNV and its WT sequence of EGFR, KRAS, and rpoB-531 gene. The error bars were standard deviations of three repetitive measurements. UV–vis absorption spectra were obtained in the range from 400 to 500 nm.

To prove the universality of this method, 11 kinds of SNV/WT DNA pairs were tested. As shown in Fig. 5, in the presence of SNV, the colorimetric signals were all obviously decreased as compared to the signal of X-probe only. What’s more, the signals just in the presence of WT were as high as X-probe only, which indicated the single base mutation could affect the strand displacement badly, resulting less G-quadruplex split and lower signal. All SNV and its corresponding WT could be discriminated, which demonstrate this approach could be adaptable for kinds of SNV detection.

## Conclusions

In conclusion, we have presented a simple and robust sensing strategy to detect SNV by naked eye. This method was based on a well-designed X-probe and a G-quadruplex. In one hand, the X-probe was a universal signal reporter without any modifications, which could improve the universality of this method and lower the cost. So the different targets could be detected by simply changing the sequences of P and C according to its target. In another hand, the X-probe enabled high specificity of probe hybridization via a toehold-mediated strand displacement. So that single base mutation could be obviously discriminated. Kinds of drug resistance-related SNVs in KRAS, EGFR and rpoB gene were successfully detected by this method, which proved its good analytical performance. This proposal was simple, rapid, low-cost, high-throughput, visible-readout, and highly specific, which have great potential for point-of-care detection.

## Methods

### Colorimetric detection procedure

Prior experiments, the strand A, B, P and C were mixed together in a 1:1:1:1 ratio with a final concentration of 0.8 μM. To formulate the X-probe, the mixture was heated in 1×TE/MgCl_2_ buffer at 95 °C water bath for 5 min, followed by slowly cooling to room temperature over the course of 90 min to avoid unwanted structure. The experiments were performed in 250 μL of solution containing 125 μL of X-probe (0.8 μM), and 125 μL of target DNA solution with different concentrations. The mixture was first incubated in TE/MgCl_2_ buffer for 60 min at 37 °C to allow toehold-mediated strand displacement. This was followed by the addition 6 μL of hemin and incubation for 30 min at room temperature, which would allow the formation of G-quadruplex/hemin complex. Finally, 250 μL of ABTS^2−^ (4 mM) and 1 μL of 4 mM H_2_O_2_ were added to the mixture and mixed completely. The absorption was collected from 400 to 500 nm on a UV-vis spectrophotometer (UV-2550, Shimadzu, Kyoto, Japan). All measurements were conducted at room temperature.

### Time-based fluorescence data acquisition

150 μL of fluorophore labeled X-probe and 150 μL of SNV or WT were mixed in the fluorescent square quartz. Then the time-based fluorescence data were obtained on a cary eclipse fluorescence spectrophotometer (Varian China co., LTD, America) with excitation at 492 nm and emission at 520 nm. All measurements were conducted at room temperature.

### Gel Electrophoresis

5 μL of the DNA solutions was mixed with 1 μL of 6× loading buffer and analyzed in 3% agarose gel electrophoresis. The electrophoresis was conducted in 1 × TBE (pH 8.0) at constant voltage of 120 V for 40 min. The gels were scanned by a UV transilluminator.

## Additional Information

**How to cite this article**: Chen, X. *et al*. A universal probe design for colorimetric detection of single-nucleotide variation with visible readout and high specificity. *Sci. Rep*. **6**, 20257; doi: 10.1038/srep20257 (2016).

## Supplementary Material

Supplementary Information

## Figures and Tables

**Figure 1:**
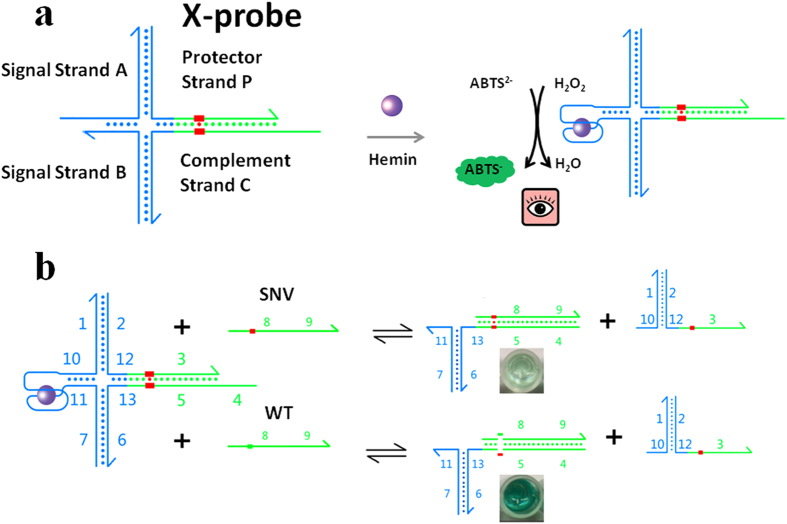
Schematic illustration of the sensing strategy for the detection of SNV based on a universal DNA probe and a split G-quadruplex.
