# Supplementary material for: A universal probe design for colorimetric detection of single-nucleotide variation with visible readout and high specificity
Source: Sci Rep. 2016 Feb 2;6:20257. doi: 10.1038/srep20257 (PMC4735751; doi:10.1038/srep20257)
Supplement: Supplementary Information [file srep20257-s1.doc]

# A universal probe design for colorimetric detection of single-nucleotide variation with visible readout and high specificity

Xueping Chen^1^, Dandan Zhou^2^, Huawei Shen^1^, Hui Chen^2^,

Wenli Feng^1^, *, Guoming Xie^1^, *

^1^Key Laboratory of Medical Diagnostics of Ministry of Education, Department of Laboratory Medicine, Chongqing Medical University, Chongqing 400016, P R China

^2^Clinical Laboratories, The First Affiliated Hospital of Chongqing Medical University, Chongqing 400016, P R China

***Correspondence authors:** Wenli Feng ([fengwlcqmu@sina.com](mailto:fengwlcqmu@sina.com)) and Guoming Xie ([guomingxie@cqmu.edu.cn](mailto:guomingxie@cqmu.edu.cn))

Table S1: Signal report strand for colorimetric detection.

| A | G TTA AAT CGT GGA TAG TAG ACG CAC ATG GGT |
| --- | --- |
| B | TGG GTA GGG CGG GTG TGC CAG GTA CAT TTG CTC GTC CTT |

Table S2: Signal report strand for fluorescence detection.

| A | BHQ1-GTG CGA ACA GGT ACA TTT GCT CGT CCT T |
| --- | --- |
| B | GTT AAA TCG TGG ATA GTA GAC TTC GCA C-FAM'6 |

Table S3: Sequences of different signal probe for optimization of G-quadruplex split modes.

| 1:1 | A | CCAAGGTGGTGTGTGTATAGTGAGGGCAGGG |
| --- | --- | --- |
|  | B | GGGAGGTGCTCACTATACACACACCACCAACC |
| 1:3+s | A | CCAAGGTGGTGTGTGTATAGTGATGGGTAGGGCGGG |
|  | B | AGTCAGTCAGTCACTCACTATACACACACCACCAACC |
|  | S | TGGGTGACTGACTGACT |
| 1:3 | A | CCAAGGTGGTGTGTGTATAGTGAATGGGT |
|  | B | TGGGTAGGGCGGGTCTCACTATACACACA CCACCAACC |

Table S4: Sequences for the optimization of the number of complement bases between A and B.

| 5’- TCG CAC | A | G TTA AAT CGT GGA TAG TAG AC TCG CAC ATG GGT |
| --- | --- | --- |
|  | B | TGG GTA GGG CGG GTG TGC GA CAG GTA CAT TTG CTC GTC CTT |
| 5’- CG CAC | A | G TTA AAT CGT GGA TAG TAG AC CG CAC ATG GGT |
|  | B | TGG GTA GGG CGG GTG TGC G CAG GTA CAT TTG CTC GTC CTT |
| 5’- G CAC | A | G TTA AAT CGT GGA TAG TAG ACG CAC ATG GGT |
|  | B | TGG GTA GGG CGG GTG TGC CAG GTA CAT TTG CTC GTC CTT |
| 5’- CAC | A | G TTA AAT CGT GGA TAG TAG AC CAC ATG GGT |
|  | B | TGG GTA GGG CGG GTG TG CAG GTA CAT TTG CTC GTC CTT |
| 5’- AC | A | G TTA AAT CGT GGA TAG TAG AC AC ATG GGT |
|  | B | TGG GTA GGG CGG GTG T CAG GTA CAT TTG CTC GTC CTT |

Table S5: Sequences of SNV, WT, and target-specific X-probe components for EGFR mutations.

| EGFR-G719A | SNV | TTCAAAAAGATCAAAGTGCTGGCCTCCGGT |
| --- | --- | --- |
|  | WT | TTCAAAAAGATCAAAGTGCTGGGCTCCGGT |
|  | P | AAGGACGAGCAAATGTACCTG CACAAAAAGATCAAAGTGCTGG |
|  | C | CGGAGGCCAGCACTTTGATCTTTTTGTG GTCTACTATCCACGATTTAAC |
| EGFR-S768I | SNV | GCCTACGTGATGGCCATCGTGGACAACCCC |
|  | WT | GCCTACGTGATGGCCAGCGTGGACAACCCC |
|  | P | AAGGACGAGCAAATGTACCTGCACTACGTGATGGCCATCGT |
|  | C | GGTTGTCCACGATGGCCATCACGTAGTGGTCTACTATCCACGATTTAAC |
| EGFR-T790M | SNV | GTGCAGCTCATCATGCAGCTCATGCCCTTC |
|  | WT | GTGCAGCTCATCACGCAGCTCATGCCCTTC |
|  | P | AAGGACGAGCAAATGTACCTGCAGCAGCTCATCATGCAGCTC |
|  | C | AGGGCATGAGCTGCATGATGAGCTGCTG GTCTACTATCCACGATTTAAC |
| EGFR-L858R | SNV | ATG TCA AGA TCA CAG ATT TTG GGC GGG CCA |
|  | WT | ATG TCA AGA TCA CAG ATT TTG GGC TGG CCA |
|  | P | A AGG ACG AGC AAA TGT ACC TGC AGT CAA GAT CAC AGA TTT TGG |
|  | C | G CCC GCC CAA AAT CTG TGA TCT TGA CTG GTC TAC TAT CCA CGA TTT AAC |
| EGFR-L861Q | SNV | TGGCCAAACAGCTGGGTGCGGAAGAGAAAG |
|  | WT | TGGCCAAACTGCTGGGTGCGGAAGAGAAAG |
|  | P | AAGGACGAGCAAATGTACCTG CAGCCAAACAGCTGGGTGCG |
|  | C | TTTCTCTTCCGCACCCAGCTGTTTGGCTG GTCTACTATCCACGATTTAAC |

Table S6: Sequences of SNV, WT, and target-specific X-probe components for KARAS mutations.

| KRAS-G12A | SNV | CTTGTGGTAGTTGGAGCTGCTGGC |
| --- | --- | --- |
|  | WT | CTTGTGGTAGTTGGAGCTGGTGGC |
|  | P | AAGGACGAGCAAATGTACCTG CAACTTGTGGTAGTTGGAG |
|  | C | GCCAGCAGCTCCAACTACCACAAGTTG GTCTACTATCCACGATTTAAC |
| KARAS-G12R | SNV | CTTGTGGTAGTTGGAGCTCGTGGC |
|  | WT | CTTGTGGTAGTTGGAGCTGGTGGC |
|  | P | AAGGACGAGCAAATGTACCTG CAACTTGTGGTAGTTGGAGC |
|  | C | GCCACGAGCTCCAACTACCACAAGTTG GTCTACTATCCACGATTTAAC |
| KARAS-G13D | SNV | CTTGTGGTAGTTGGAGCTGGTGACGTAGGC |
|  | WT | CTTGTGGTAGTTGGAGCTGGTGGCGTAGGC |
|  | P | AAGGACGAGCAAATGTACCTG CATGTGGTAGTTGGAGCTGG |
|  | C | CTACGTCACCAGCTCCAACTACCACATG GTCTACTATCCACGATTTAAC |
| KARAS-G13V | SNV | CTTGTGGTAGTTGGAGCTGGTGTCGTAGGC |
|  | WT | CTTGTGGTAGTTGGAGCTGGTGGCGTAGGC |
|  | P | AAGGACGAGCAAATGTACCTG CATGTGGTAGTTGGAGCTGG |
|  | C | CTACGACACCAGCTCCAACTACCACATG GTCTACTATCCACGATTTAAC |
| KARAS-Q61H | SNV | GCAGGTCACGAGGAGTACAGTGCAATGAGG |
|  | WT | GCAGGTCAAGAGGAGTACAGTGCAATGAGG |
|  | P | AAGGACGAGCAAATGTACCTG CAAGGTCACGAGGAGTACAG |
|  | C | TCATTGCACTGTACTCCTCGTGACCTTG GTCTACTATCCACGATTTAAC |

Table S7: Sequences of SNV, WT, and target-specific X-probe components for EGFR mutations.

| rpoB-531 | SNV | ACCCACAAGCGCCGACTGTTG |
| --- | --- | --- |
|  | WT | ACCCACAAGCGCCGACTGTCG |
|  | P | AAGGACGAGCAAATGTACCTG CA ACCCACAAGCGCCGA |
|  | C | CAACAGTCGGCGCTTGTGGGTTGGTCTACTATCCACGATTTAAC |

Table S8: Sequences for the mismatched detection.

| rpoB-531 | Target DNA | ACCCACAAGCGCCGACTGTTG |
| --- | --- | --- |
|  | Single-base mismatch DNA | ACCCACAAGCGCCGACTGTCG |
|  | Three-base mismatch DNA | ACCCACAAGCGCCGACTCACG |
|  | Non-complementary DNA | TAGTGGTCTCATGTCCACGTA |
| EGFR-T790M | Target DNA | GTGCAGCTCATCATGCAGCTCATGCCCTTC |
|  | Single-base mismatch DNA | GTGCAGCTCATCACGCAGCTCATGCCCTTC |
|  | Three-base mismatch DNA | GTGCAGCTCATCTCACAGCTCATGCCCTTC |
|  | Non-complementary DNA | TACTGATGACCAGTCGACGAACATGATCGT |
| KARAS-G12R | Target DNA | CTTGTGGTAGTTGGAGCTCGTGGC |
|  | Single-base mismatch DNA | CTTGTGGTAGTTGGAGCTGGTGGC |
|  | Three-base mismatch DNA | CTTGTGGTAGTTGGAGCAGCTGGC |
|  | Non-complementary DNA | TACTGATGTCCACTCTAGGAACTA |

Table S9: Comparisons of this work with previously reported methods.

| List | Method | Read-out | Labeling | Analysis time(h) | Discrimination factor | Reference |
| --- | --- | --- | --- | --- | --- | --- |
| 1 | Protected DNA strand displacement for enhanced single nucleotide discrimination in double-stranded DNA | Fluorescence | No | 2.5 | 5% | 1 |
| 2 | Gold nanoparticle enhanced fluorescence anisotropy for the assay of single nucleotide polymorphisms (SNPs) based on toehold-mediated strand-displacement reaction | Fluorescence | Yes | 1.5 | 1% | 2 |
| 3 | Colorimetric detection of single nucleotide polymorphisms in the presence of 10^3^-fold excess of a wild-type gene | Colorimetric | Yes | 2.0 | 0.1% | 3 |
| 4 | Highly effective colorimetric and visual detection of nucleic acids using an asymmetrically split peroxidase DNAzyme | Colorimetric | No | 1.5 | 5% | 4 |
| 5 | Fluorescence-based detection of single-nucleotide changes in RNA using graphene oxide and DNAzyme | Fluorescence | Yes | 2.0 | 1% | 5 |
| 6 | Gold-nanoparticle-based colorimetric discrimination of cancer-related pointmutations with picomolar sensitivity | Colorimetric | Yes | 1.5 | Unknown | 6 |
| 7 | Enzyme-mediated single-nucleotide variation detection at room temperature with high discrimination factor | Fluorescence | Yes | 1.5 | 0.05% | 7 |
| This work | A universal probe design for colorimetric detection of single-nucleotide variation with visible readout and high specificity | Colorimetric | No | 1.5 | 0.05% |  |

Fig. S1: The absorbance at 418 nm for the detection of mismatch DNA in rpoB, EGFR-T790M and KRAS-G12R.


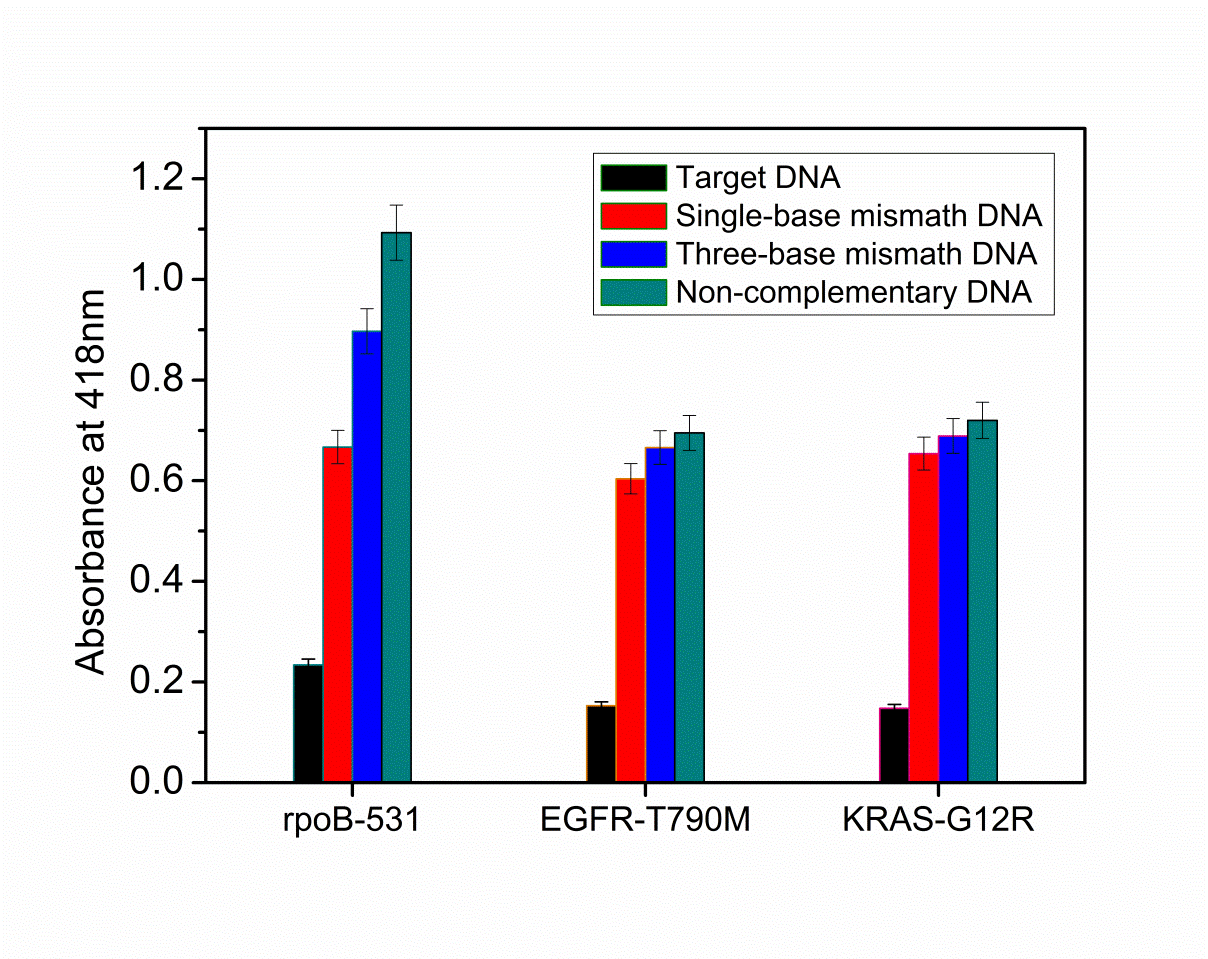


**References**

1. [Wang, D](http://www.ncbi.nlm.nih.gov/pubmed/?term=Wang D%5BAuthor%5D&cauthor=true&cauthor_uid=22830619)., [Tang, W](http://www.ncbi.nlm.nih.gov/pubmed/?term=Tang W%5BAuthor%5D&cauthor=true&cauthor_uid=22830619)., [Wu, X](http://www.ncbi.nlm.nih.gov/pubmed/?term=Wu X%5BAuthor%5D&cauthor=true&cauthor_uid=22830619). et al. Highly selective detection of single-nucleotide polymorphisms using a quartz crystal microbalance biosensor based on the toehold-mediated strand displacement reaction. [*Anal. Chem*.](http://www.ncbi.nlm.nih.gov/pubmed/22830619) 84, 7008-7014 (2012).

2. [Wang, X](http://www.ncbi.nlm.nih.gov/pubmed/?term=Wang X%5BAuthor%5D&cauthor=true&cauthor_uid=23062556)., [Zou, M](http://www.ncbi.nlm.nih.gov/pubmed/?term=Zou M%5BAuthor%5D&cauthor=true&cauthor_uid=23062556)., [Huang, H](http://www.ncbi.nlm.nih.gov/pubmed/?term=Huang H%5BAuthor%5D&cauthor=true&cauthor_uid=23062556). et al. Gold nanoparticle enhanced fluorescence anisotropy for the assay of single nucleotide polymorphisms (SNPs) based on toehold-mediated strand-displacement reaction. [*Biosens. Bioelectron*.](http://www.ncbi.nlm.nih.gov/pubmed/23062556) 41, 569-575 (2013).

3. Deng, H, Shen, W, Gao, Z. et al. Colorimetric detection of single nucleotide polymorphisms in the presence of 10³- fold excess of a wild-type gene. [*Biosens.Bioelectron*.](http://www.ncbi.nlm.nih.gov/pubmed/23062556)  68, 310-315 (2015)..

4. Deng, M., Zhang, D., Zhou, Y. & Zhou, X. Highly effective colorimetric and visual detection of nucleic acids using an asymmetrically split peroxidase DNAzyme. *J. Am. Chem. Soc.* 130, 13095-13102 (2008).

5. Hong, C. et al. Fluorescence-based detection of single-nucleotide changes in RNA using graphene oxide and DNAzyme. *Chem. Commun.* 51, 5641-5644 (2015).

6. Valentini, P. et al. Gold-nanoparticle-based colorimetric discrimination of cancer-related point mutations with picomolar sensitivity. *ACS Nano.* 7, 5530-5538 (2013).

7. Wu, T., Xiao, X., Zhang, Z. & Zhao, M. Enzyme-mediated single-nucleotide variation detection at room temperature with high discrimination factor. *Chem. Sci.* 6, 1206-1211 (2015).
